# Our Whole Lives for Hypertension and Cardiac Risk Factors—Combining a Teaching Kitchen Group Visit With a Web-Based Platform: Feasibility Trial

**DOI:** 10.2196/29227

**Published:** 2022-05-16

**Authors:** Paula Gardiner, Lisa McGonigal, Ariel Villa, Lara C Kovell, Pallavi Rohela, Andrew Cauley, Diana Rinker, Barbara Olendzki

**Affiliations:** 1 Department of Family Medicine and Community Health University of Massachusetts Chan Medical School Worcester, MA United States; 2 Division of Cardiovascular Medicine Department of Medicine University of Massachusetts Chan Medical School Worcester, MA United States; 3 Department of Population and Quantitative Health Sciences University of Massachusetts Chan Medical School Worcester, MA United States

**Keywords:** hypertension, health disparities, teaching kitchen, technology, mindfulness, low income, medical group visits, mobile phone

## Abstract

**Background:**

Hypertension (HTN) affects millions of Americans. Our Whole Lives: an eHealth toolkit for Hypertension and Cardiac Risk Factors (OWL-H) is an eHealth platform that teaches evidence-based lifestyle strategies, such mindfulness and cooking skills, to improve self-management of HTN.

**Objective:**

The primary goal of this pilot study was to evaluate the feasibility of OWL-H combined with teaching kitchen medical group visits (TKMGVs) in a low-income population of participants with HTN.

**Methods:**

We conducted a pre-post 8-week study to assess the feasibility of a hybrid program (a web-based 9-module self-management program, which includes mindfulness and Mediterranean and Dietary Approaches to Stop Hypertension diet) accompanied by 3 in-person TKMGVs among patients with HTN. Data including demographics, platform use, and satisfaction after using OWL-H were examined. Outcome data collected at baseline and 8 weeks included the Mediterranean Diet Questionnaire, Hypertension Self-Care Profile Self-Efficacy Instrument, Blood Pressure Knowledge Questionnaire, and the number of self-reported blood pressure readings. For the statistical analysis, we used descriptive statistics, paired sample *t* tests (1-tailed), and qualitative methods.

**Results:**

Of the 25 enrolled participants, 22 (88%) participants completed the study. Participants’ average age was 57 (SD 12.1) years, and 46% (11/24) of them reported a household income <US $30,000 per year. Among the 22 participants who logged in to OWL-H, the average number of mindfulness practices completed was 7 and the average number of module sessions accessed was 4. In all, 73% (16/22) of participants reported that they were “very satisfied” with using OWL-H to help manage their HTN. Participants’ blood pressure knowledge significantly increased from baseline (mean 5.58, SD 1.44) to follow-up (mean 6.13, SD 1.23; *P*=.03). Participants significantly increased their adherence to a Mediterranean diet from baseline (mean 7.65, SD 2.19) to follow-up (mean 9, SD 1.68; *P*=.004). Participants’ self-efficacy in applying heart-healthy habits, as measured by the Hypertension Self-Care Profile Self-Efficacy Instrument, increased from baseline (mean 63.67, SD 9.06) to follow-up (mean 65.54, SD 7.56; *P*=.14). At the 8-week follow-up, 82% (18/22) of the participants had self-reported their blood pressure on the OWL-H platform at least once during the 8 weeks.

**Conclusions:**

The eHealth platform for HTN self-management, OWL-H, and accompanying in-person TKMGVs have the potential to effectively improve lifestyle management of HTN.

**Trial Registration:**

ClinicalTrials.gov NCT03974334; https://clinicaltrials.gov/ct2/show/NCT03974334

## Introduction

Hypertension (HTN) affects millions of Americans, with a disproportionate burden falling on both those within the lowest socioeconomic status (SES) demographic and on people of color [1]. Currently, to increase access and self-efficacy, there is a heightened need for technology targeted toward delivery of HTN self-management strategies. However, few studies have tested the use of internet-delivered self-management interventions within lower SES populations [2-7].

HTN is a complex disease, with both external and internal factors affecting its control, including healthy diet, stress management, and medication use [8]. From the patient’s standpoint, it requires an understanding of basic physiology and the concept that one’s behavior (dietary habits, physical activity, and stress) has an impact on blood pressure levels [9]. Although treatment options such as dietary and behavior change counseling can increase healthy dietary habits that can reduce HTN, only 35% of adults with HTN receive such interventions [10]. These factors, coupled with external factors such as food insecurity; decreased access to fruits and vegetables and locations to exercise; and high-stress jobs, likely play an important role in significantly poor HTN control [8,11-14].

Adults are increasingly seeking dietary information from internet sources and apps, and these digital health tools present scalable ways to improve lifestyle changes [15,16]. Furthermore, as of 2019, up to 71% of Americans who earned <US $30,000 owned a smartphone and 56% had home broadband service [17]. However, there has been little research conducted with low-income individuals accessing web-based tools to improve HTN self-management [18,19]. Before embarking on a large-scale trial, appropriate methodologies must be adapted or developed and tested for feasibility in a target population consisting of individuals with HTN and other cardiac risk factors and low SES. Therefore, this study examines the feasibility of a web-based multimodal self-management platform called Our Whole Lives: an eHealth Toolkit for Hypertension and Cardiac Risk Factors (OWL-H), combined with a teaching kitchen medical group visit (TKMGV) in a low-income population.

Originally adapted from mindfulness-based stress reduction [20-22], the Our Whole Lives (OWL) web-based platform was created as a patient-derived web-based self-management toolkit named Our Whole Lives: an eHealth Toolkit for Chronic Pain (version 1). It was created in 2014 as part of a Patient Centered Outcomes Research Institute randomized controlled trial for patients with chronic pain and depression. OWL was designed with a patient advisory group and beta-tested with diverse groups to ensure that patients from varied racial, ethnic, generational, and low–health literacy backgrounds could comprehend its information [20]. The OWL curriculum introduces patients to the principles of mindfulness practices, nutrition, stress reduction, and movement [22,23]. In 2017, OWL (version 2) was tested to determine its effectiveness as a stand-alone intervention for patients with chronic pain; participants showed significant reduction in depression, pain interference, and average pain impact, accompanied by 13% reduction in opioid use (*P*=.03) [24].

**Figure 1:**
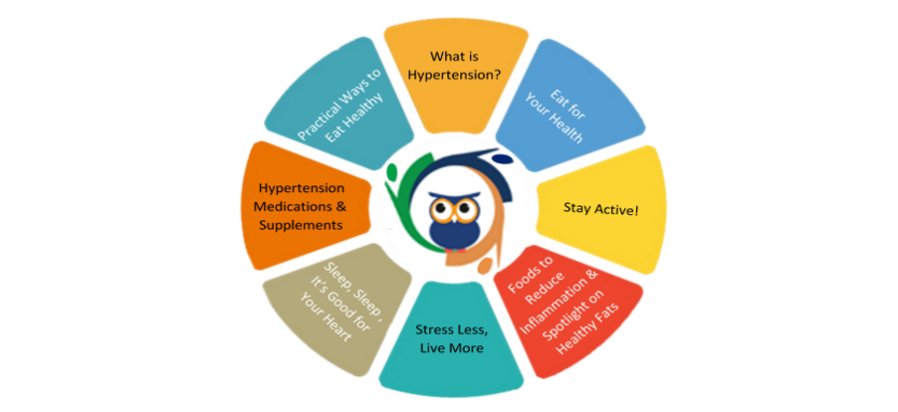
Our Whole Lives: an eHealth toolkit for Hypertension and Cardiac Risk Factors patient curriculum diagram.

Using the proven OWL platform and with funding from the University of Massachusetts Medical School’s Center for Advancing Point of Care Technologies in Heart, Lung, Blood, and Sleep Diseases, this pre-post study was powered to test the feasibility (though not the clinical outcomes) of OWL (version 3), Our Whole Lives for Hypertension and Cardiac Risk Factors. This was combined with accompanying in-person medical group visits and hands-on cooking classes in a teaching kitchen (ClinicalTrials.gov NCT03974334). Using the principles of adult education, the new adapted platform provides content and experiential activities to increase self-management of HTN through regular blood pressure monitoring and self-management strategies to reduce cardiac risk factors such as sodium consumption, stress, and unhealthy eating. Figure 1 shows the OWL-H curriculum on the platform.

## Methods

### Setting

This study was conducted at the University of Massachusetts Memorial Health Care system, which provides access to the large and diverse patient population in central and western Massachusetts. The city of Worcester, Massachusetts, itself allows access to a diverse local population, with >13% of the city’s residents being Black or African American, 7% being Asian, and 21% being Hispanic or Latino, with 21% of the population living below the poverty line [25].

### Study Design

This feasibility trial enrolled participants with a current diagnosis of HTN to test OWL-H. Totally, 2 cohorts of patients (N=24) participated in an 8-week clinical trial testing of hybrid live cooking classes or medical group visits, combined with a web-based self-management platform (OWL-H). Participants’ qualitative comments and feedback were used to revise the OWL-H platform for the next iteration.

### Recruitment and Enrollment

Inclusion criteria were as follows: people with a current diagnosis of HTN who were aged >18 years, could provide informed consent, and can understand educational materials written in English and whose physical and mental health status were sufficient to comprehend instructions and participate in the interventions. Participants also had to have access to computer technology (mobile phone, desktop, or laptop) and the internet to use the web-based OWL-H platform. Exclusion criteria were as follows: people with serious underlying comorbid physical, cognitive, or psychiatric disease, including psychotic or manic symptoms, which precluded full participation in the intervention; those involved in active substance abuse; pregnant women or those who were actively trying to become pregnant; and those not willing to participate in the intervention or attend medical group visits. In addition, those planning to begin new HTN treatments within 2 weeks from the beginning of the protocol or who were planning a major medical treatment within 4 months from being screened were not eligible to participate.

Participants were recruited from the Worcester area with a focus on adult primary care clinics and were either self-referred (via flyers and brochures distributed around the clinics) or referred by their primary care providers. They spoke in person or over the phone with the study research coordinator (RC) to complete the self-report portion of the screening process and review the study objectives and eligibility criteria. If eligibility was confirmed, the study RC read a study consent fact sheet to the participant and obtained verbal consent.

### Intervention Web-Based Platform

OWL-H is a password-protected website hosted on a Health Insurance Portability and Accountability Act–compliant server that can be accessed via a computer, tablet, or mobile device. It contains an orientation session (for training purposes) and 8 unique educational sessions (Figure 1; Table 1). One session is released each week, across 8 weeks, and participants can access these unlocked sessions at any time [26-31]. The study team (licensed dietician or nutritionist [BO], a culinary-trained family medicine physician [LJM], and a mindfulness-trained family physician [PG]) adapted the content from OWL (version 2) to create OWL-H. Most of the original content of OWL (version 2) created for patients with chronic pain was used (Table 1 outlines the new content that was added to strategies to reduce HTN) [24]. The third version added evidence-based self-management strategies for reducing HTN and cardiac risk factors such as cooking videos and recipes using the Dietary Approaches to Stop Hypertension (DASH) [32,33] and Mediterranean diets. The new content developed specifically for OWL-H was completed with the input of patients in the clinic, who were attending a medical group visit at University of Massachusetts Memorial Health Care, to ensure appropriate literacy levels and diverse patient engagement. In addition, curriculum changes were made based on participant feedback between cohort 1 and cohort 2.

**Table 1:** Summary of the OWL^a^ website and the in-person teaching kitchen medical group visit.

Week and title of the session			Theme or activity		Home practice and recipes
**1**					
	Teaching kitchen 1–micronutrients=building blocks^b^	Sodium, potassium, and calcium and changing tastes; introduction to fiber and healthy carbohydrates; eating more fruits, veggies, herbs and spices; drinking more water; knife skills and safety; and basic cooking skills		Pear, spinach, and walnut salad, salsa, oil-free pesto, chia seed pudding, and water bar	
	OWL–orientation to our group	Awareness of breath M^c^, ground rules, introduction to mindfulness, and video on how to measure BP^d^ (3.54 minutes)		Engage group members on community tab and set up home practice space	
	OWL–what is HTN^e^?^b^	Summary of HTN, how HTN is measured and treated, introduction to HTN management lifestyle habits^b^, introduction to BS, and video on what is HTN? (12 minutes)		Self-monitoring BP, journal^b^, and BS^f^ on 6 out of 7 days	
2	OWL–eat for your health^b^	Introduction to DASH^g^ and Mediterranean eating plans (video 17.52 minutes)^b^ and the healthy plate method and video on introduction to M		Eat one meal mindfully, share healthy plate pictures^b^, BS on 6 out of 7 days, and M on 6 out of 7 days	
3	OWL stay active^b^	Setting SMART^h^ movement goals, exercise education and guidance^b^ introduction to MM^i^, and video on stay active (12:30 minutes)		Come up with a 3 day/week exercise plan^b^ and alternate BS and MM on 6 out of 7 days	
**4**					
	Teaching Kitchen 2–what do we have with our veggies tonight?^b^	Fats-saturated, omega 3s; protein and all its sources (plant based); fiber; sugar; traffic light foods, glycemic index, hunger, and portion control; alcohol and other enjoyments; vegetable entrees; and what is “mise en place”?		Three-bean salad, roasted chickpeas, avocado ice cream, and zucchini noodles	
	OWL–foods to reduce inflammation and spotlight on healthy fats^b^	Nonpharmacological approaches to treating inflammation, tips to reduce salt intake and increase healthy fat consumption^b^, video on inflammation (4:07 minutes), and spotlight on salt (4:36 minutes)		Post a recipe on the community tab, create a plan to substitute salt for spices^b^, and alternate BS and M on 6 out of 7 days	
5	OWL–stress less, live more	Nonpharmacological approaches to reducing stress and video on our reaction to stress (4:08 minutes)		Create a stress reduction plan, alternate BS and MM, and M on 6 out of 7 days	
6	Sleep, sleep, it’s good for your heart	Nonpharmacological approaches to sleep and video on the importance of healthy sleep (5:15 minutes)		Create healthy sleep space and share plan on the community tab, alternate BS and MM, and loving kindness M on 6 out of 7 days	
7	OWL-HTN medications and supplements^b^	Discussion of common classes of HTN medications and common side effects, research-supported supplements for HTN^b^ introduction to loving kindness M, and videos on vitamins and minerals (4:08 minutes) and food as pharmacy (19:38 minutes)		Notice any physical sensations when taking the prescribed BP medications^b^ and choice of BS, MM, M, or loving kindness M on 6 out of 7 days	
**8**					
	OWL–practical ways to eat healthy^b^	A review of important food groups for patients with HTN, tips for grocery shopping and eating out at restaurants^b^, and video on practical eating (8:21 minutes)		Create a heart-healthy plate shopping list and share on the community tab^b^	
	Teaching kitchen 3–cooking *competition*^b^	Putting it all together; eating out–how to order; meal planning; planning ahead–batch cooking and freezing; shopping list–staples for your pantry; budget cooking and shopping; reading nutrition labels; trying new foods; and cooking *competition*		Quick lemon and garlic quinoa salad; carrot and beet salad; Asian salad; cocoa banana smoothie; spinach, cucumber, and mint smoothie; and kale, coconut, and pineapple smoothie	

^a^OWL: Our Whole Lives.

^b^Though all sessions’ pictures, formatting, and content were updated in some way, this denotes brand new content created specifically for OWL for Hypertension and Cardiac Risk Factors.

^c^M: meditation.

^d^BP: blood pressure.

^e^HTN: hypertension.

^f^BS: body scan.

^g^DASH: Dietary Approaches to Stop Hypertension.

^h^SMART: Specific, Measurable, Achievable, Realistic, and Timely.

^i^MM: mindful movement.

Upon logging in to OWL-H, participants were greeted with a 4-item *check-in* box asking them to rate their mood (ranging from 0-10, with high numbers indicating positive mood) and physical comfort (ranging from 0-10, with high numbers indicating high levels of comfort); record their current blood pressure and pulse; and answer whether they had taken their high blood pressure medication today, if applicable. Within OWL-H, the measurement record charted these daily readings throughout the duration of the program so that participants and staff can track their progress. All the participants received an Omron 7 Series upper-arm blood pressure monitor with an appropriately sized cuff.

After completing the check-in process, participants had the option to navigate through OWL-H session modules, tools, or resource pages. Each session included 1-2 videos on health topics, with duration ranging from 2-20 minutes, covering the week’s topic (Figure 1). Next, the participants completed a home practice (eg, meditation, body scan, and mindful movement) and posted it on the community board or journal. When any media item was played and completed, a comment box appeared to facilitate collection of real-time qualitative feedback from the participants (“What are your thoughts and feelings after completing this video?”).

Home practices, assigned at each session, included mindful movement (a video with duration of 24 minutes or audio with duration of 32 minutes), meditation with a focus on the breath (3 audio options with duration of approximately 20 minutes each), and body scans with a focus on noticing sensations in different areas of the body (3 audios with duration of 23 minutes or 12 minutes). Each mind–body practice was recorded by a certified yoga or meditation teacher. As participants progressed through the sessions, they were slowly introduced to new techniques or longer meditations to enhance their experience, and after completion of each, they were asked to provide feedback on the session. The mind–body progress log, which can be accessed from within the session or the upper taskbar, added a gamification element to OWL-H by tracking the completion of these practices. The participants received 1 checkmark and puzzle piece to fill in their week’s progress on the log. By practicing for 6 out of 7 days, the participants can earn enough puzzle pieces to complete the week’s reward image.

**Figure 2:**
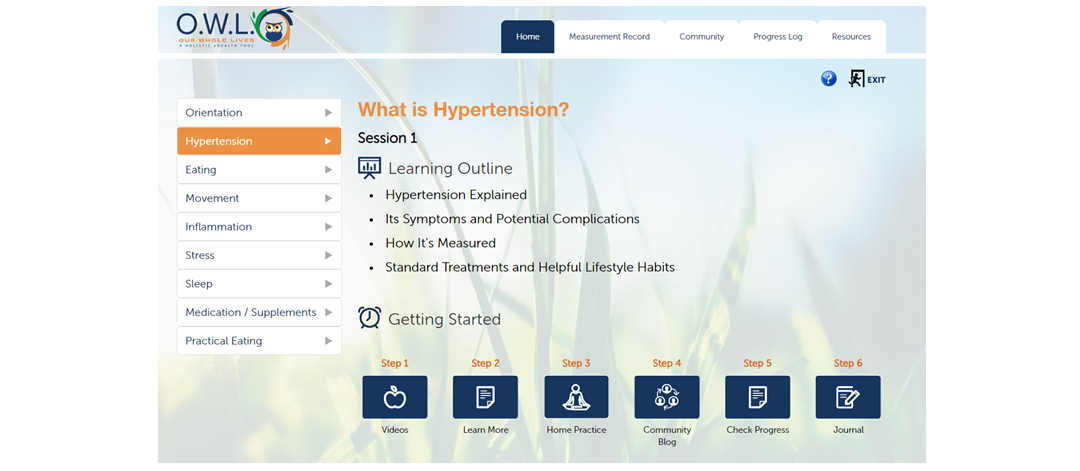
Our Whole Lives: an eHealth toolkit for Hypertension and Cardiac Risk Factors (OWL-H) version 3 platform.

The journal is a private feature that allows participants to write about their daily events, thoughts, and reactions at their own time. In addition, the home practices periodically asked the participants to follow up on a lesson learned that week through a written journal assignment, such as writing about common stresses or creating a healthy shopping list. Participants were asked to participate in the monitored web-based discussions hosted on the community board of the website. OWL-H’s extensive resource library contains 12 unique content areas, all derived through participant feedback and requests (focus on health, mind and body, poems, quotes, nutrition resources, nutrition tips, healthy eating, recipes, integrative medicine, support services, videos, and audios). Each area contains information and tools that support what is learned in the main session content, either directly linked from within a session’s written materials or as stand-alone supplemental material. It also contains information on local resources (eg, affordable gyms, food pantries, free meals, and community resources), spotlights on foods of interest (eg, salt, whole grains, fruit, and vegetables), information on mindfulness and mental health, and motivational content (eg, poems; Figure 2) [33-37].

### Overview of In-Person TKMGV

#### TKMGV Session 1 (Week 1)

**Figure 3:**
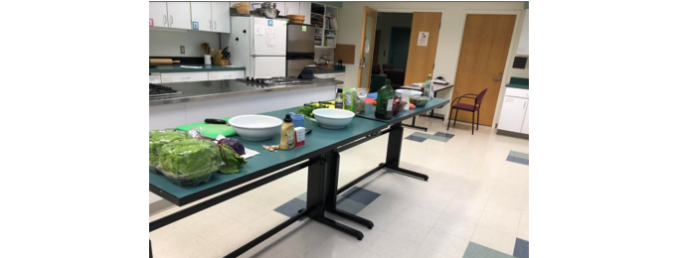
Teaching kitchen next to group visit room.

During the first in-person session, all participants were welcomed to the room and asked to sit in a circle and complete an individual medical check-in form before meeting the clinician one-on-one in a side room. In a group setting, participants were taught to use an Omron Series 7 upper-arm cuff that was lent to them for the duration of the study and were provided a binder containing the cooking class recipes, their OWL-H log-in information, user manual, printed resource documents, and a welcome letter. The participants were trained in and observed measuring their own blood pressure. Next, the study staff assisted participants in accessing and bookmarking OWL-H on the device of their choice (ie, smartphone, tablet, or laptop). Participants then logged in to the OWL-H for the first time, following a live demonstration of the website by the study staff. The remainder of the session was conducted in the teaching kitchen in the next room (Table 1; Figure 3). The recipes and cooking classes were facilitated by a culinary-trained physician (LM). One week after this first class, the RC called each participant to assess whether there were any issues with using OWL-H and to answer any questions.

#### TKMGV Session 2 (Week 4)

For week-4 TKMGV, the program check-in structure remained the same, and clinicians led an in-person interactive presentation on the DASH and Mediterranean diet. Once the check-in and teaching-kitchen portion was completed, the participants reviewed any technical issues or questions they had about OWL-H.

#### TKMGV Session 3 (Week 8)

For the final TKMGV, the program check-in structure was the same as the first class: (1) participants completed the postintervention outcomes and feedback surveys, and physical outcome measures were collected by the study staff and (2) after completing the cooking portion of TKMGV, participants could stay and participate in an optional focus group to facilitate further collection of qualitative feedback; all in attendance chose to do so. Participants could receive US $50 in the form of an Amazon gift card for completing all aspects of the study, US $25 for completing the 8-week program and all questionnaires, and another US $25 for participating in the optional focus group. All the participants who completed the study also received a certificate of completion.

### Self-report Outcome Data Collection

As a feasibility study, the primary outcomes were centered around confirming whether OWL-H is a feasible tool to deliver and facilitate self-management of HTN and other cardiac risk factors. The second primary outcome was to characterize participants’ use of and feedback on OWL-H to further adapt and revise the content and platform for use in a large study. These data were collected through surveys administered at baseline and 8 weeks (or web-based through REDCap [Research Electronic Data Capture; version 9.3.0; Vanderbilt University] [38] if a participant was not able to attend in person). Questions regarding participants’ preintervention and postintervention blood pressure monitoring habits were asked, and a blood pressure entry log was collected using the OWL-H system to provide quantifiable self-management data. Using these outcomes, we evaluated preintervention and postintervention effects.

Demographic information included the following: age, sex, race, ethnicity, country of birth, marital status, health insurance status, education level, employment status, and yearly household income. Other questions assessed the status of social and cardiac determinants of health such as food security (“In the last 12 months, the food that I bought just didn’t last, and I didn’t have the money to get more” and “In the last 12 months, I couldn’t afford to eat balanced meals”), access to healthy food (“In the last 12 months, I couldn’t afford to buy fresh fruit and vegetables”), prescribed blood pressure medication (“Do you currently take a prescribed blood pressure medication?”), whether they smoke cigarettes or drink alcohol (“How often do you practice non-smoking [tobacco]?” and “How often do you practice moderation in drinking alcohol daily [2 glasses or less for men; 1 glass or less for women]?”), and their recent level of stress (“Stress means a situation in which a person feels tense, restless, nervous, or anxious, or is unable to sleep at night because of his or her mind is troubled all the time. Within the last 30 days, how often have you felt this kind of stress?”).

The Blood Pressure Knowledge Questionnaire (BPKQ) [39,40] was used to assess the participants’ level of knowledge regarding risk factors of HTN. It consists of eight items covering topics such as common symptoms, white coat syndrome, and the amount of salt consumed by the average American individual, with a mix of multiple-choice (6 items) and true or false questions (2 items). The total number of correct items determines the score, which ranges from 1 to 8, with high scores indicating high level of knowledge. The information needed to answer each item correctly was covered in the OWL-H’s curriculum.

The Hypertension Self-Care Profile (HTN-SCP) consists of three separate instruments designed to assess self-care behavior, motivation for self-care, and self-efficacy in people with HTN. It was developed and validated in 2014 [41]. The self-efficacy instrument (HTN-SCP–Self-Efficacy [HTN-SCP–SE]) used in this study consisted of 20 items assessing the respondent’s confidence, on a scale of 1 (not confident) to 4 (very confident), to regularly engage in lifestyle habits that are recommended for patients with high blood pressure. This resulted in a total confidence or self-efficacy score ranging from 20 to 80, with high scores indicating high levels of confidence in engaging in heart-healthy lifestyle habits.

The Mediterranean Diet Questionnaire consists of 14 yes or no items that assess the participant’s consumption of heart-protecting or heart-harming foods, such as various healthy proteins (eg, legumes, nuts, and fish) and commercial sweets or pastries, respectively. It was originally developed in Spain in 2004 to briefly assess adherence to a Mediterranean diet [42] for patients at risk for cardiac complications and has been validated in multiple studies [43]. Items assess not only the type of food consumed but also whether the appropriate serving size is consumed, on a weekly basis. The number of “yes” responses are totaled, resulting in a score ranging from 0 to 14, with high scores indicating high adherence to the Mediterranean diet.

To assess the blood pressure self-monitoring before and after the intervention, we asked the following question at baseline, “Are you regularly (at least once per week) able to measure your blood pressure outside of your doctor appointments?” Those who said “yes” were asked, “How often, in a regular week, do you measure your blood pressure outside of your doctor appointments?” In their postintervention surveys, they were again asked to estimate their average weekly number of entries. Along with the self-reported numbers, the OWL-H platform also maintains a log of every blood pressure reading entered by each participant across the 8 weeks. Surveys were administered either in person, over the phone, or via an email invitation through REDCap—a password-protected research tool.

### OWL System Outcome Data

Self-reported blood pressure and pulse data were collected each time the participants logged in. Collected data included participant’s use of the OWL-H website, such as the number of minutes spent on the site, use of home practices, or number of health videos completed; any text that was inputted in the website through the comment boxes, journals, and community board; and any resource content that was accessed.

### Qualitative Data Collected on OWL-H

Qualitative data were gathered in real time through written comments in media comment boxes. Qualitative feedback was also collected from the participant entries into the journal and through surveys on both the specific sessions and the overall OWL-H content and platform. The video comments and journal entries were collected to gain an understanding of reflections, reactions, and opinions about the OWL-H project and to ascertain their impact on the overall satisfaction of the patients.

### Data Analysis

Descriptive statistics were used to analyze the survey information. Means, SDs, frequencies, and percentages were calculated for demographic characteristics. To compare the results between baseline and follow-up, we used paired sample *t* tests (1-tailed) and descriptive statistics. We carried the missing values forward from the pretest measurements to account for missed survey items. Means and SDs were calculated for the BPKQ, Mediterranean Diet Questionnaire, HTN-SCP–SE, and blood pressure self-monitoring at baseline and 8 weeks. All quantitative analyses were conducted using SPSS (version 26, IBM Corp).

For OWL use data, we tracked and summed the average and total number of accessed mind–body practices, number of times the participants used the journal, and total duration of time spent on OWL-H.

Qualitative data analysis methods were used to identify themes that were related to participants’ OWL-H platform use. The media comments and journal entries were reviewed and independently coded by 2 research assistants (LCK and AC). Using modified grounded theory, the coders inductively generated new codes. After the media comments and journal entries were coded, the 2 research assistants (LCK and AC) reconciled the codes with the RC and resolved any differences through consensus and review. The coders identified high-density codes and combined similar codes into categories and themes, which were shared with the research team.

### Ethics Approval

This study was approved by the University of Massachusetts Medical School institutional review board (H00015619).

## Results

### Overview

**Figure 4:**
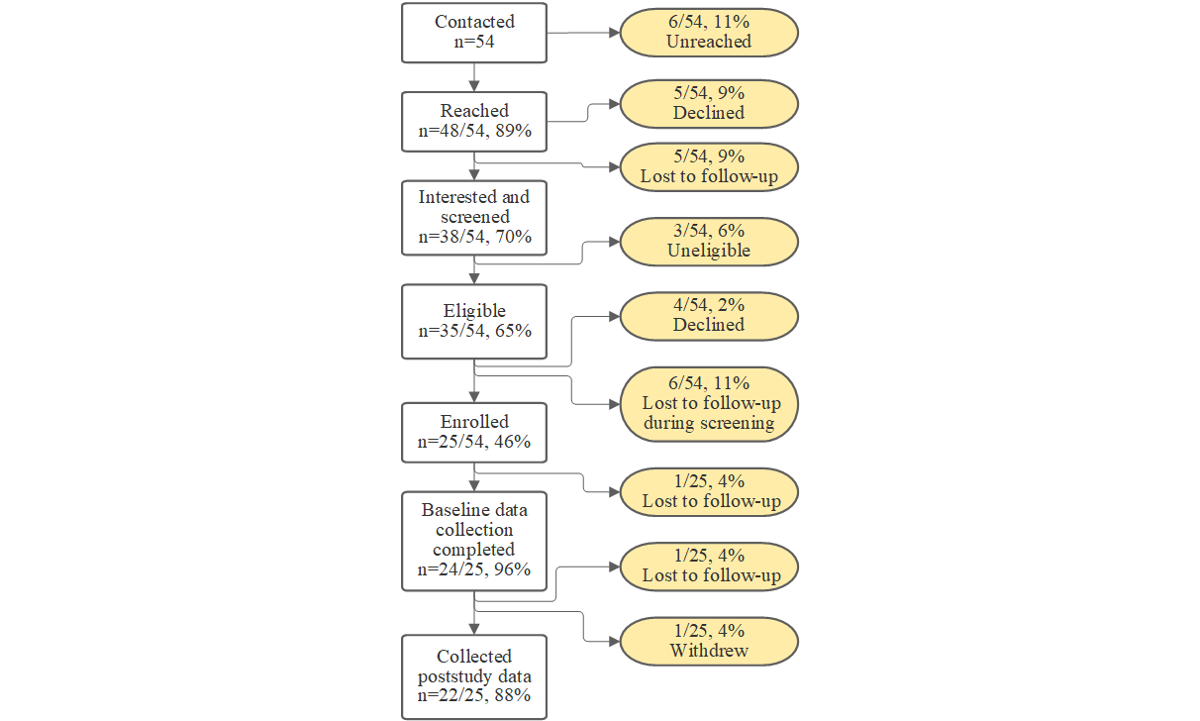
Study enrollment CONSORT (Consolidated Standards of Reporting Trials) diagram.

The study flow, screening, and study enrollment are illustrated in Figure 4.

The study team contacted a total of 54 candidate participants of which 38 (70%) participants agreed to be screened (3/38, 8% participants were screened and found ineligible), 35 (65%) participants were eligible, and 25 (46%) participants consented. After enrollment and consent (25/54, 46%), 4% (1/25) of the participants voluntarily withdrew from the study and 8% (2/25) of the participants were lost to follow-up (1 before baseline data collection; N=24). Of the 24 participants, 22 (92%) participants completed follow-up data collection. Specifically, in cohort 1, there were 42% (10/24) of participants in the beginning and 41% (9/22) of participants at the end, whereas for cohort 2, there were 63% (15/24) of participants in the beginning and 59% (13/22) of participants in the end.

Table 2 lists all the demographic factors. In this study, the average age was 57 (SD 12.1) years, most participants were women (21/24, 88%), and 33% (8/24) of participants identified as belonging to a race other than White, and 17% (4/24) of participants identified as Hispanic. In all, 46% (11/24) of participants’ annual household incomes were <US $30,000, with 79% (19/24) of participants reporting they “have just enough money to make ends meet” or “do not have enough money to make ends meet or prefer not to answer” on a monthly basis. Participants responded “often true or sometimes true” that “the food that I bought just didn’t last, and I didn’t have the money to get more” (7/24, 29%); that they “couldn’t afford to eat balanced meals” (8/24, 33%); and that they “couldn’t afford to buy fresh fruits and vegetables” (9/24, 38%). Totally, 46% (11/24) of participants reported having used a food insecurity service (eg, Supplemental Nutrition Assistance Program; Women, Infants, and Children program; and food pantries).

**Table 2:** Demographic characteristics (N=24).

Variables			Baseline values
Age (years), mean (SD)			57 (12.1)
Sex (women), n (%)			21 (88)
**Race, n (%)**			
	White		16 (67)
	Other^a^		8 (33)
Hispanic or Latino (yes), n (%)			4 (17)
**Education level, n (%)**			
	Up to high-school diploma or general education development		3 (13)
	Some college, no degree		12 (50)
	Bachelor degree or higher		9 (38)
**Employment status, n (%)**			
	Working outside the home		12 (50)
	Unemployed or retired		10 (42)
	Student or home maker		2 (8)
**Yearly household income (US $), n (%)**			
	0-29,999		11 (46)
	≥30,000		11 (46)
	Prefer not to answer		2 (8)
**Country of birth, n (%)**			
	United States		17 (71)
	Outside the United States		7 (29)
**Marital status, n (%)**			
	Married		12 (50)
	Not married		12 (50)
**Health insurance type, n (%)**			
	Public		12 (50)
	Private		12 (50)
Blood pressure (baseline systolic diastolic; mm Hg), mean (SD)			131 (17)/88 (11); (minimum 110/68, maximum 167/123)
**In general, how do your finances usually work out at the end of the month? Do you find that you usually...? n (%)**			
	End up with some money left		5 (21)
	Have just enough money to make ends meet		14 (58)
	Do not have enough money to make ends meet or prefer not to answer		5 (21)
**In the last 12 months...** **, n (%)**			
	**Often true or sometimes true**		
		...the food that I bought just didn't last, and I didn't have the money to get more.	7 (29)
		...I couldn't afford to eat balanced meals.	8 (33)
		...I couldn't afford to buy fresh fruit and vegetables.	9 (38)
	**Never true**		
		...the food that I bought just didn't last, and I didn't have the money to get more.	17 (71)
		...I couldn't afford to eat balanced meals.	16 (67)
		...I couldn't afford to buy fresh fruit and vegetables.	15 (63)
**Food insecurity services^b^ (collapsed), n (%)**			
	Have not used a food insecurity service		13 (54)
	Have used at least one food insecurity service		11 (46)
**Stress means a situation in which a person feels tense, restless, nervous, or anxious, or is unable to sleep at night because of his or her mind is troubled all the time. Within the last 30 days, how often have you felt this kind of stress?^c^, n (%)**			
	None of the time		4 (17)
	A little of the time		7 (29)
	Some of the time		7 (29)
	Most of the time or all the time		5 (21)

^a^Other races include Asian or Pacific Islander (1/8, 13%), Black or African American (3/8, 38%), other (2/8, 25%), prefer not to answer (1/8, 13%), and >1 race (1/8, 13%).

^b^Food insecurity service includes Supplemental Nutrition Assistance Program or food stamps (7/11, 64%); Women, Infants, and Children program (5/11, 45%); food pantries (4/11 36%); community free meals (1/11, 9%); and Church programs (1/11, 9%).

^c^A total of 1 response was missing.

### Survey Outcomes

Table 3 lists the baseline and follow-up mean values and comparisons for all the outcomes. Participants’ HTN knowledge, measured using the BPKQ, significantly increased from baseline (mean 5.58, SD 1.44) to follow-up (mean 6.13, SD 1.23; *P*=.03). Participants’ self-efficacy in applying heart-healthy habits, as measured by the HTN-SCP–SE, increased from baseline (mean 63.67, SD 9.06) to follow-up (mean 65.54, SD 7.56), but there was no significant change (*P*=.14). Participants showed a significant increase in their adherence to a Mediterranean diet from baseline (mean 7.65, SD 2.19) to follow-up (mean 9, SD 1.68; *P*=.004).

**Table 3:** Survey outcome measures (N=24).

Items	Baseline total sample, n (%)	Baseline value, mean (SD)	Postintervention total sample, n (%)	Postintervention value, mean (SD)	*t* test (*df*)	*P* value
Blood Pressure Knowledge Questionnaire	24 (100)	5.58 (1.44)	22 (92)	6.13 (1.23)	−2.07 (21)	.03
Hypertension Self-Efficacy Questionnaire	24 (100)	63.67 (9.06)	22 (92)	65.54 (7.56)	−1.13 (21)	.14
Mediterranean Diet Questionnaire	23 (96)	7.65 (2.19)	22 (92)	9 (1.68)	−2.96 (21)	.004

At baseline, of the 24 participants, 10 (42%) participants reported being able to measure their blood pressure at home. For those 10 participants, the mean of the self-reported baseline number of blood pressure measurements per week was 2.8 (SD 1.31; minimum=1, maximum=5). At the 8-week follow-up, 82% (18/22) of the participants had reported their blood pressure in OWL-H at least once during the 8 weeks. The self-reported average weekly blood pressure measurement of these participants was 4.78 (SD 1.90; minimum=1, maximum=7), with the OWL-H platform data reporting the mean weekly entries as 2.74 (SD 2.02). Across 8 weeks, the mean total number of measurements logged was 22.22 (SD 16.54; minimum=1, maximum=54). When considering only the participants who completed the entire 8-week protocol (22/24, 92%), the mean total log of blood pressure was 24.05 (SD 16.14; minimum=1, maximum=54). When dividing the total number of measurements logged for the same 92% (22/24) of the participants across the 8 weeks, the weekly logged blood pressure mean was 3 (SD 2.02). There was no difference in the change between baseline blood pressure 131/88 mm Hg (minimum=110/68 mm Hg, maximum=167/123 mm Hg; SD 17 systolic pressure and SD 11 diastolic pressure) and 8-week blood pressure 134/84 mm Hg (minimum=104/58 mm Hg, maximum=160/103 mm Hg; SD 16 systolic pressure and SD 11 diastolic pressure).

Participants’ average mood score across the 8 weeks was 7.23 (SD 1.70) and average physical comfort score was 6.63 (SD 2.11). After accounting for those who did not complete the study (2/24, 8%) and those not taking blood pressure medication (2/24, 8%), the average adherence to taking a prescribed blood pressure medication across the 8 weeks was 85% (17/20).

**Figure 5:**
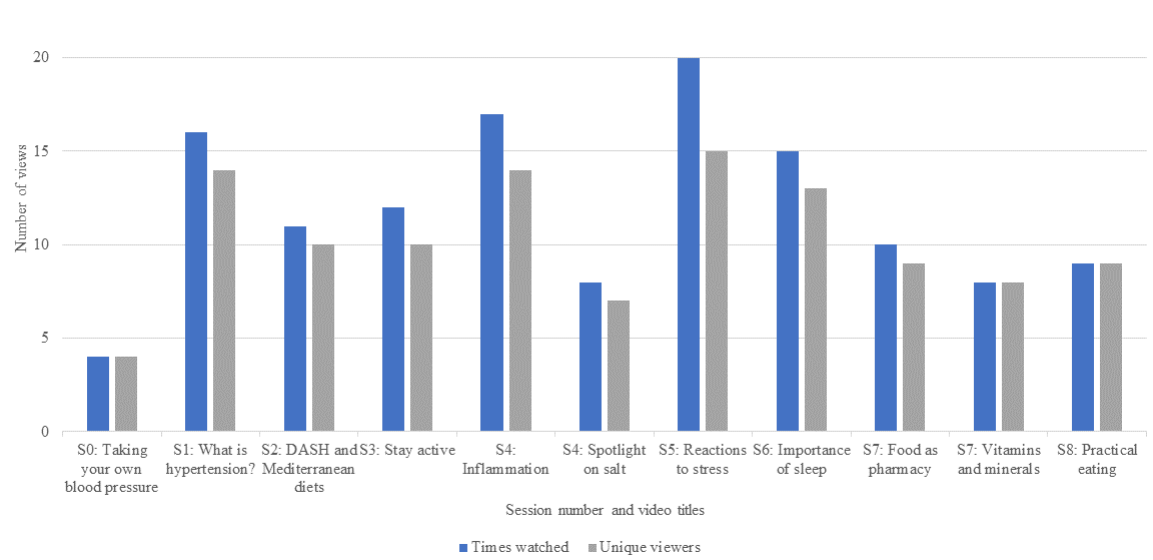
Summary of completed video views by session (S). DASH: Dietary Approaches to Stop Hypertension.

Of the 9 modules, the average number of modules accessed was 4 (minimum=0, maximum=9). The average number of unique educational documents accessed from the resource library was 8.27 (minimum=0, maximum=51). Of the 24 participants, 11 (46%) participants attended all 3 cooking classes, whereas 3 (13%) participants attended only 1 class. Of the 24 participants, 2 (8%) participants did not spend time on OWL-H after the orientation class, and the remaining 22 (92%) participants used OWL-H on their own at least once. For those 22 participants, the average number of minutes spent in using the site was approximately 30 hours or 1773.64 minutes (minimum=101 minutes, maximum=4789 minutes). Participants’ average number of mindfulness home practices completed was 7 (minimum=0, maximum=40) and average number of completed videos on health topic was 7 (minimum=0, maximum=22). In terms of participation in mind–body practices at home, on average, participants completed 3 body scans (minimum=0, maximum=16), 2 meditations (minimum=0, maximum=18), and 1 yoga session (minimum=0, maximum=12). Further analysis of platform use is shown in Figure 5 (video use). These results show a trend of decreased use over time (Multimedia Appendix 1 reports mind–body activity schedule and Multimedia Appendix 2 reports mind–body activity use rates).

Overall, participant satisfaction and feedback about OWL-H was positive. From the survey feedback, we found that 73% (16/22) of the participants reported that they were “very satisfied” with using OWL-H to help manage their HTN. In all, 73% (16/22) of the participants reported that it was “very helpful” in “helping [you] to take and record [your] blood pressure at home.” Totally, 82% (18/22) of participants said that they “feel like OWL provided support and encouragement to reach [your] goals,” 73% (16/22) of them said that they would “like to use OWL again,” and 82% (18/22) of them said that they “would recommend OWL to someone they know.” Further participant feedback is presented in Textbox 1.

The main themes that emerged from the content feedback were centered on the health benefits of the mind–body practices, self-reflection on healthy behaviors, behavior change, and nutrition knowledge and skills (Textbox 1). Participants who practiced the body scans, meditations, or mindful movements overwhelmingly felt beneficial effects on their health. Of the 22 participants, 11 (50%) participants expressed that the practices had effects on their stress, pain levels, or blood pressure. Specifically, regarding blood pressure, a participant remarked, “Very effective. Dropped my systolic 15 pts.”

After watching the videos or participating in home practices, most participants (13/22, 59%) reflected on their own behaviors and beliefs. Videos on health topics such as *Practical Eating* and *Stay Active* had participants writing about their current or past health behaviors, and often ended with them deciding to reinforce a health habit, make a change, or discuss barriers to change. Participants said that they learned a variety of new nutrition skills to help them make healthy choices, such as “I will try ginger root tea. I will introduce turmeric into my cooking more as well. I could add a bit of flaxseed to my steel cut oats too.”

A mild adverse event was reported during the study; however, it was determined to be unrelated to the study.

**Summary of qualitative Our Whole Lives for Hypertension and Cardiac Risk Factors media content (eg, videos and audios) feedback.**

**Theme and original quotes**

- Participants who practiced the body scans, meditations, or mindful movement activities overwhelmingly felt beneficial effects on their health, such as stress reduction, pain awareness and reduction, and actual decline in measured blood pressure.
  “I found the meditation very relaxing and calming and definitely something I would use as a tool to relieve some stress- which I do. I breathe in and out and it seems to help a great deal” [participant 1].
  “Yesterday I fell and hurt my knee and back. So today when I did the body scan it helped with locating my exact pain locations, so I was able to relax those areas to cut down the pain” [participant 2].
  “Tried taking BP before and after body scan. Before: 165/92 After: 113/53 That’s really impressive” [participant 3].
- After watching videos or practicing activities, many participants reflected on their own behaviors and beliefs or on how the material has impacted their lives.
  “I totally love her voice and the meditation. Sometimes people get angry at family members, but that is just a waste of energy. So sending them love will make you feel better” [participant 4].
- Participants found that the material in the videos and practices encouraged them to try healthy behaviors or reinforced their already healthy behaviors.
  “I really enjoyed listening to this video. I already have put into practice much of the tips that were shared, however, I learned a few new things too. Things like cutting more veggies and fruits than needed, looking at the top and bottom of supermarket shelves to find the best bargains, and looking at the UNIT price of an item to see if I am really saving money. I knew about staying on the outer perimeters of markets for finding healthier choices too. This program has really changed the way I think about food and I have seen great results in the weight I have lost and my blood pressure medication being reduced. Thank God for this program!!” [participant 5].
- Patients learned a variety of new health and nutrition knowledge and skills to help them make healthy lifestyle choices.
  “I really liked how the doctor explains high blood pressure (HBP) in easy terms what it is, so anyone can understand. I didn’t know about the mm of mercury being the standard measurement for it, for everyone. There are many devastating heart problems associated with HBP, and I often forget that if not controlled, could lead to a stroke, heart attack and so forth. But the good news is, to be aware it truly is the silent killer. My mother had three a heart attacks, and one of my sisters had one, and had stents put in. Knowledge is power to change my life. Thankful for the doctor and her team, to teach me, and eager to learn new healthier ways to lower my HBP” [participant 6].

## Discussion

### Principal Findings

The importance of using technology to encourage self-management and self-monitoring for patients with cardiac risk factors and HTN has become even more important during the COVID-19 pandemic, especially for those with health disparities in access to high blood pressure treatment [44]. We recruited participants with HTN to engage with the OWL-HTN platform combined with attending a TKMGV and found that 73% (16/22) of participants were highly satisfied with using OWL-H to manage their HTN. Participants’ blood pressure knowledge and adherence to a Mediterranean diet significantly increased. There was an increase in self-efficacy in applying heart-healthy habits. In all, 82% (18/22) of the participants reported their blood pressure in OWL-H at least once during the 8 weeks.

During the pandemic, the importance of internet access and health delivery technologies to assist with self-monitoring and social connection, such as mobile phones, computers, and tablets, has increased [17,45]. For example, in a recent study by Lustria et al [46], the prevalence of internet use was 72% among those with self-reported HTN or diabetes, which translates to 63 million US adults. Technologies present a way to create a community, educate, and sustain engagement in health practices. However, patients with low income and experiencing high blood pressure may not have access to the internet and spaces to exercise and have increased stress and food insecurity, further exacerbating disparities in HTN risk [47-49]. Furthermore, low diet quality is even more prevalent among adults with food insecurity, and diet quality is directly related to morbidity and mortality of cardiovascular diseases [47,49-51]. Therefore, it is important that this population have increased patient education and skills, such as healthy shopping and cooking, stress reduction, and regular monitoring of their blood pressure.

Studies have shown a link between knowledge about HTN and blood pressure outcomes [52]. Insufficient knowledge about HTN could lead to less optimal blood pressure control through lower rates of adherence to prescribed medications and engagement in lifestyle practices [53-57]. We found that the participants’ knowledge of common blood pressure and cardiac risk factors significantly increased after the intervention. High knowledge of HTN was associated with healthy lifestyle practices, including eating smaller portions to lose weight and reducing dietary sodium. Abu et al [58], in a study of adults with HTN who attended 2 primary care clinics in the city of Baltimore, Maryland, found that patients with low knowledge about HTN were less likely to be engaged in heart-healthy lifestyle practices than patients with high knowledge about HTN.

Both the Mediterranean dietary pattern and the DASH diet have been shown to improve blood pressure in adults and have been linked to reduced cardiac injury [59-62]. Our intervention presented engaging content, cooking demonstrations using a teaching kitchen, and recipes from the Mediterranean and DASH diets (high in fruits, vegetables, whole grains, and lean proteins, and low in red meats, sweets, saturated, and total fat) [63] and we showed an increase in adherence to a Mediterranean diet. There is very little research on the use of teaching kitchen or culinary skills as an intervention to reduce blood pressure [64-66]. In a study by Razavi et al [67], families living in New Orleans, Louisiana, were randomized to a hands-on teaching kitchen culinary education class or non–kitchen-based dietary counseling for 6 weeks. Compared with families receiving traditional dietary counseling, those participating in hands-on kitchen-based nutrition education were approximately 3 times as likely to follow a Mediterranean dietary pattern [67]. Qualitative findings from OWL emphasized themes that included healthy shopping on a budget, cooking one’s own food, and adapting the DASH and Mediterranean diets to appeal to diverse ethnic groups and income levels.

There is strong evidence that mind–body techniques such as meditation are linked to low blood pressure [68-70]. The main themes that emerged from the video content feedback were centered on the health benefits of the mind–body practices, self-reflection on healthy behaviors and beliefs, behavior change or reinforcement of healthy behaviors, and new health and nutrition knowledge and skills (Textbox 1). Participants who practiced the body scans, meditations, or mindful movements overwhelmingly felt beneficial effects on their health. In addition, the most widely viewed videos were on the topics of stress and inflammation, 2 videos assigned after the second in-person TKMGV; hence, more research is needed on how patients interact with live or web-based curriculum and which component leads to behavior change.

We found that participants increased their blood pressure monitoring. At the 8-week follow-up, 82% (18/22) participants had reported their blood pressure in OWL-H at least once during the 8 weeks. In the Framingham Health eHeart study, women were more likely than men to enroll in a digital intervention that tracked weekly blood pressure and activity with a Fitbit device, with high rates of device use through the 5-month intervention [71,72]. We showed that it is feasible to deliver the intervention at a teaching kitchen as part of a medical group visit and use web-based tracking, content, and experiences (eg, meditation) to reinforce self-management of HTN. Our future research direction includes a large fully powered randomized controlled trial that will allow us to test which parts of the intervention have the greatest impact or whether there is synergy in the different components that lead to behavior change.

### Limitations

As a feasibility pilot study, the sample size was not powered to detect reductions in blood pressure. External factors such as the variability in internet connectivity and type of device, such as smartphones, computers, or tablets, may have impacted the results. No valid methodology exists to measure and account for these external factors. In addition, the engagement of male participants was low (3/24, 13%), a limitation reported in other studies of similar nature [73-75]. The future upgrade of OWL-H will aim to include study materials in non-English languages with a goal to increase efficacy of the intervention for ethnically diverse participants.

### Conclusions

As these technologies are associated with healthy behaviors, they present a way to create a community, educate, and sustain engagement in health. The flexible access to remote learning platforms creates a convenient and cost-efficient way to reach patients who are under geographic, pandemic, and economic burdens that prevent health care access.

## Appendix

Multimedia Appendix 1 Mind–body activity schedule.

Multimedia Appendix 2 Mind–body activity use rates.

## Abbreviations

BPKQ: Blood Pressure Knowledge Questionnaire
DASH: Dietary Approaches to Stop Hypertension
HTN: hypertension
HTN-SCP–SE: Hypertension Self-Care Profile Self-Efficacy
OWL: Our Whole Lives
OWL-H: Our Whole Lives: an eHealth toolkit for Hypertension and Cardiac Risk Factors
RC: research coordinator
REDCap: Research Electronic Data Capture
SES: socioeconomic status
TKMGV: teaching kitchen medical group visit
