# Supplementary figures and images for: Our Whole Lives for Hypertension and Cardiac Risk Factors—Combining a Teaching Kitchen Group Visit With a Web-Based Platform: Feasibility Trial
Source: JMIR Form Res. 2022 May 16;6(5):e29227. doi: 10.2196/29227 (PMC9152723; doi:10.2196/29227)

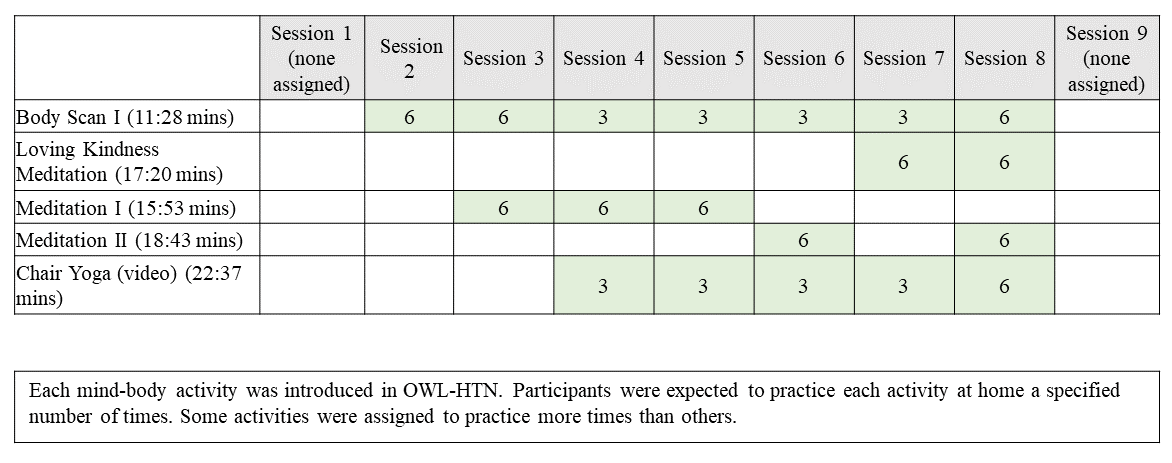

Supplement: Multimedia Appendix 1 [file formative_v6i5e29227_app1.png]

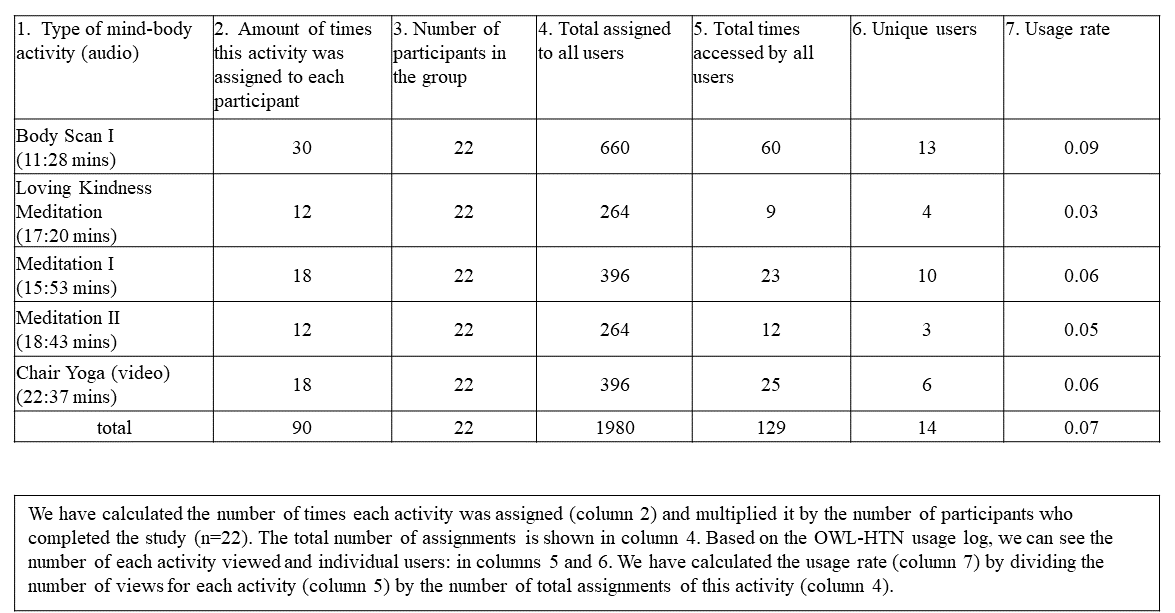

Supplement: Multimedia Appendix 2 [file formative_v6i5e29227_app2.png]
